# Engaging Youth and Young Adults in the COVID-19 Pandemic Response via the “It’s Our Turn” Crowdsourcing Contest

**DOI:** 10.3390/ijerph20065112

**Published:** 2023-03-14

**Authors:** Linnea A. Evans, Omar Gomez, Dulce J. Jiménez, Heather J. Williamson, Ann Turnlund Carver, Sairam Parthasarathy, Samantha Sabo

**Affiliations:** 1 Department of Health Promotion and Policy, University of Massachusetts, Amherst, MA 01003, USA; 2 Center for Health Equity Research, Northern Arizona University, Flagstaff, AZ 86011, USA; 3 Southwest Interdisciplinary Research Center, Arizona State University, Phoenix, AZ 85004, USA; 4 Division of Pulmonary, Allergy, Critical Care and Sleep Medicine, University of Arizona, Tucson, AZ 85724, USA

**Keywords:** COVID-19, adolescents and young adult health, youth engagement in research, crowdsourcing, art, mental health, health equity

## Abstract

As the coronavirus disease (COVID-19) pandemic continued to progress into 2021, appeals were made to take a stronger focus on the perceptions and practices of youth and young adults (YYAs) regarding COVID-19 mitigation, as well as the impact of mitigation strategies on the overall wellbeing of YYAs. In this paper, we describe our efforts to increase YYA engagement in Arizona’s COVID-19 response by pairing embedded values from youth participatory action research (YPAR) with a crowdsourcing challenge contest design. The research protocol and implementation are described, followed by a thematic analysis of YYA-led messaging portrayed in 23 contest submissions and reflections formed by 223 community voters after viewing contest submissions. The authors conclude that a YYA-led crowdsourcing contest presented an opportunity to (a.) investigate the perceptions and behaviors of YYAs and their networks regarding the COVID-19 pandemic and mitigation efforts *and* (b.) amplify the voices of YYAs in the pandemic response. Perhaps even more importantly, this approach also offered insight into the exacerbated impact of the pandemic on YYA mental health and wellbeing, and the utility of YPAR in raising awareness of these effects among the contexts and social networks of YYAs.

## 1. Introduction

By the spring of 2021, public health researchers and practitioners were well into a year of contending with coronavirus disease (COVID-19) misinformation and efforts to increase public engagement in COVID-19 mitigation strategies, and yet, youth and young adult (YYA) engagement was still underdeveloped in the pandemic response. As the return to school neared for the 2021–2022 academic year, appeals were made to take a stronger focus on the perceptions and practices of YYAs regarding COVID-19 mitigation, as well as the impact of mitigation strategies on their overall—both mental and physical—wellbeing [1]. In this paper, we describe our efforts to increase YYA engagement in Arizona’s COVID-19 response by combining principles embedded in youth participatory action research (YPAR) with crowdsourcing approaches that emphasize group-based problem solving. First, we detail our logic for prioritizing YYAs in community-engaged research that aims to increase COVID-19 awareness and reflects the needs and experiences of Arizonan YYAs. To this end, we employed power-sharing principles [2] by integrating a diverse YYA advisory group to co-plan and execute the ‘It’s Our Turn’ crowdsourcing contest. This contest challenged YYAs to try their own hand at creating a health message about COVID-19 prevention or raise awareness about ways the pandemic had affected YYAs. Contest submissions were widely circulated and voted upon by community members, which further served to disseminate YYA-led messages that were top-of-mind for YYAs. Research protocols are detailed, followed by a thematic analysis of YYA-led messaging portrayed in contest submissions, and reflections formed by community voters after viewing the contest submissions. 

### 1.1. Why Engage Youth and Young Adults (YYAs) in COVID-19 Pandemic Research?

Interest in youth and young adults (YYAs) grew when surveillance and research efforts showed that COVID-19 infections could also result in severe health effects among this age group, including long COVID symptoms, multisystem inflammatory syndrome, and even death [3,4,5]. Concerns over the health consequences of YYA infections, however, were being weighed against incertitude for vaccine safety among this age group. Even into the fall of 2021, a third of parents said they would not get their 12–17-year-old teen vaccinated, despite vaccines first being available for older youth in late 2020 and 12–15-year-olds in May 2021 [6]. Slower vaccine uptake among YYAs illuminated the need to better understand the perceptions of YYA and their networks around COVID-19 vaccination in order to better tailor mainstream prevention messaging [1]. 

YYA engagement in research also draws upon the change-making capabilities possessed by YYAs. Younger generations have often been at the forefront of social change movements, leading student sit-ins during the civil rights movement; and more recently championing LGBTQ+ equality, protesting police brutality, and challenging world leaders to act on climate change [7,8,9]. On smaller-scale health campaigns, youth-led efforts have proven effective in creating behavioral change among peers [10,11,12]; reducing chronic disease risk factors among adult family and community members [13,14]; and altering the institutional policies of municipal government and educational systems [15,16,17]. Certain sectors of adult society may be more receptive to youth-led messaging than those led by government authorities [18,19,20]. As such, the persuasion of YYAs on their networks may be particularly strategic when seeking shifts in perceptions and behaviors on health topics steeped in stigma or political mistrust, such as COVID-19 mitigation and other pandemic-related health impacts (e.g., mental health). 

### 1.2. Best Practices in Youth-Engaged Research, and Pandemic-Necessitated Adaptations

Participatory approaches are central to research that generates questions, methods, and outcomes aligned with the needs, contexts, and preferred communication style of the very communities most affected by social, political, and economic issues [21,22]. For example, in youth participatory action research (YPAR), principles of best practice include the pursuit of inquiry-based topics that are grounded in youths’ lived experiences and concerns; genuine collaboration that builds upon the expert knowledge and skills of youth; and opportunities for youth to take on leadership positions that actively intervene to create social change [23]. The execution of these principles involves power-sharing between YYAs and academic researchers, while iteratively integrating research and actions for social change [2,22].

Prior to the COVID-19 pandemic, most YPAR initiatives relied on in-person meetings to foster power-sharing and a bi-directional exchange of knowledge, e.g., [24,25]. In pandemic times, however, the need to socially distance and rapidly respond to changing contexts necessitates adaptations in the execution of YPAR frameworks. For example, during the pandemic, virtual meetings were supplanting in-person methods for YYA engagement, typically considered the best practice in YPAR [26,27]. Social distancing practices also require innovation in transformative actions that youth can undertake without collectively gathering in-person to mobilize change. One such innovation may be found in crowdsourcing initiatives, an approach that solicits solutions to an identified problem via a large group of people, often through the use of social media and alternative forms of communication. 

### 1.3. Crowdsourcing as an Approach to Increasing Community Engagement in Health Research

Crowdsourcing refers to a distributed problem-solving model whereby an organization solicits solutions via open calls to a large group of people [28,29,30]. Crowdsourcing techniques have been employed in marketing, public policy, and more recently, health research and promotion initiatives [31,32,33]. A hallmark feature of crowdsourcing is regard for the power of group-based thinking that draws upon varied forms of knowledge and expertise, in contrast to the dominant practice of raising up solutions offered by a small subset of academically trained experts [34]. Thus, in theory, crowdsourcing approaches align with the power-sharing principles emphasized in YPAR, guiding the conduct of research operations in a manner inclined towards health equity [30]. 

One crowdsourcing technique gaining traction in health research is contest challenges [32,34,35,36]. The typical implementation of a crowdsourcing contest involves organizing a steering committee to advise on contest implementation; issuing an open call to the public for new ideas specific to a particular health issue; evaluating received submissions via community vote and/or expert judges; public celebration of finalists’ entries via prizing; and amplifying dissemination of received content, often through the platforms of partnering organizations [30]. Open calls to challenge contests can be issued across varied communication mediums, such as in-person events, flyers, radio, and social media platforms. As such, challenge contests may mitigate many of the barriers that constrain community participation, such as static meeting times, transportation and childcare needs, and the unfortunate strain Brown and Black people often endure when entering or interacting with institutions that have a history of devaluing communities of color [37]. In pandemic times, these barriers become even more exaggerated, making the use of crowdsourcing contests an appropriate consideration when attempting to engage the community in COVID-19 research. 

Given the alignment between crowdsourcing challenge contests and power-sharing principles emphasized in YPAR, we selected a youth-led crowdsourcing contest as our approach to engaging the voices and creativity of YYAs in COVID-19 health messaging research. Next, we will describe the research protocol of the “It’s Our Turn” crowdsourcing contest, followed by the results evaluating contest entries and community reach. 

## 2. Materials and Methods

### 2.1. The Arizona Community Engagement Alliance (AZ-CEAL) against COVID-19

In the fall of 2020, the National Institutes of Health (NIH) funded the Arizona Community Engagement Alliance (AZ-CEAL) Against COVID-19 to undertake community-engaged research focused on understanding perceptions and behaviors related to COVID-19 mitigation among three of the racial/ethnic groups hardest hit by COVID-19: Native, Hispanic/Latinx, and Black/African-American communities [38]. The Arizona CEAL COVID Consortium (AC3) is comprised of four academic institutions, Arizona State University (ASU), Northern Arizona University (NAU), the University of Arizona (UA), and the Mayo Clinic (Mayo), in partnership with the Arizona Community Health Workers Association (AzCHOW; the statewide network of community health workers), local health departments, federally qualified health centers, and faith and community-based organizations. Additionally, through this network, a collaboration was formed between the CEAL team and the Maricopa branch of the NAACP, which ultimately led to their co-sponsorship on youth engagement activities. Research activities of the AZ-CEAL program were approved by the University of Arizona Institutional Review Board (IRB Protocol# 2011244240), and written consent and assent for contest participation and voting were obtained from the youth (<18 years of age) as well as the parent or legally authorized representative (parent/caregiver).

### 2.2. Formation of a Youth and Young Adult Advisory Board 

In July 2021, the AZ-CEAL team released a call to youth-based networks to recruit YYAs, age 16–25 years, to serve as YYA advisors to the research. Recruitment outlets included summer camps (e.g., high school health clubs, Indigenous Scholars Program), school networks, sporting teams, public libraries, and social media. In an effort to achieve diverse representation by racial and ethnic identity, age, gender, geographical location, and knowledge base and skill set, project staff asked YYA applicants to share their demographics, motivations for applying, and their unique abilities when completing a short application administered through the Qualtrics survey platform. In total, the AZ-CEAL received 84 complete applications and selected 14 YYA advisors to guide the contest launch, data collection, and dissemination of messaging conveyed in the artwork contest submissions. 

Given the high rates of COVID-19 community transmission and the pause on in-person events, our research team knew that most of our engagement activities would need to occur virtually. While a virtual environment enhanced our ability to simultaneously interact with YYAs from across the state, it also imposed a challenge in establishing rapport between adult researchers and YYA advisors, as well as between YYA team members. As such, we used multiple modes of engagement to build rapport. For example, prior to our first meeting, all team members were invited to complete a Google JamBoard, an interactive whiteboard where users can draw or add pictures, shapes, and text about the things they love to do, and the people and places important to them—colloquially referred to as ‘their jam’. The JamBoard compilation became a low-stakes, creative way to learn about one another. 

Four virtual Zoom meetings were held between August and October 2021 to orient YYA advisors to the CEAL initiative and explore their thoughts on a crowdsourcing concept. All meetings were scheduled in the late afternoon. In the few instances where a YYA advisor could not make the group meeting time, they were encouraged to share their input asynchronously via email, phone, or one-on-one Zoom meeting. YYA expertise was sought on all aspects of the action-based research, including the overarching study concept and design, recruitment materials and outlets, contest awards, data collection, and dissemination efforts. For instance, YYAs shaped the scope of inquiry by selecting the contest name, “It’s Our Turn”, and recommending an open-endedness to the types of messaging received. As such, we incorporated the following language in contest recruitment flyers: “Share your creativity to help protect Arizona communities from COVID-19 and raise awareness on how the pandemic affects youth”. This approach solicited contributions befitting of the funding agency’s priorities (e.g., vaccine uptake) *and* the experiences and priority needs of YYAs. Additionally, YYA advisors made recommendations for the contest website and flyer to include the use of bright colors and large text; simplifying language and length of consent forms; revisions to the question stems and response options on data collection accompanying the contest implementation; and appropriate prizes for contest winners. Remuneration for their time and effort was made to each advisor via a $250 gift card. 

### 2.3. Contestant Recruitment Strategies and a Platform to Solicit “It’s Our Turn” YYA Crowd-Sourced Messaging 

Feedback from the YYA advisory board informed the development of a customized website where ‘the YYA crowd’ could enter their contest submissions, along with print and electronic flyers to direct interest to the contest webpage. The content included information on the “It’s Our Turn” contest goal, rules for submission, and contest prizes (materials available upon request). The contest was launched in September 2021, inviting YYA Arizonans, age 16 to 25 years (later expanding eligibility to ages 14–15 years), to submit pandemic-related messaging important to them. YYAs were directed to choose their own unique approach (e.g., TikTok or YouTube video, photography, drawing, song/rap, podcast) to create messaging on COVID-19 vaccination and masking, or to raise awareness on challenges youth are facing during the pandemic. Prize incentives for first-time entry were USD 10 gift card per participant and USD 250 gift card for the 12 winners selected by Arizonans. 

In addition to the design of recruitment materials, YYA advisors led community outreach efforts to promote the contest in their school, youth-based clubs, social media platforms, and other public spaces in which they frequently interact (e.g., coffee shops, workplaces, and tribal lands). YYA advisors tracked their recruitment efforts in real-time via Google Excel (e.g., advisor name, city and flyer placement, number of social media views, and interactions with interested YYA). 

Institutional review board approval was sought to collect data from contestants to evaluate YYA perceptions and priorities on COVID-19 mitigation strategies, pandemic effects, and the overall effectiveness of this approach to YYA engagement. As such, a Qualtrics online survey tool link was embedded within the contest webpage to administer a web-based informed consent and collect the artistic submission of contestants, the motivation behind their submission entry, along with basic demographics and vaccination status. Parental/guardian consent was required for contestants under 18 years of age. 

### 2.4. Dissemination of YYA-Crowdsourced Health Messaging via Community Vote, Presentations, and Public Display of YYA Art

As a first step in the dissemination of YYA-crowdsourced health messages, an open call was issued to the public to vote for the top finalists. Contest submissions were posted to the contest website and organized into 4 categories, intrinsically derived from entry style: visual arts, public service announcement, poetry/written word, and toolkit. Voters were instructed to select their top pick in each category, for a total of 12 finalists (ages 14–15 were judged separately from 16+ years). Similar to the contestant data collection protocol, a Qualtrics online survey tool link was embedded within the contest webpage to administer web-based informed consent and data collection from voters age 14 and older. In addition to collecting their top vote for each of the stylistic categories, voters were asked basic demographic questions and to reflect on the following: (1.) the reason for their vote (open-ended question format) and (2.) whether viewing the stylistic category influenced perceptions and behavioral practices relevant to COVID-19 mitigation or made them think more about how the pandemic has affected youth (closed-ended format). 

Promotion of the community vote occurred via YYA advisors, AZ-CEAL partners, and a press release circulated to youth- and community-based organizations and local news outlets. In response to the press release, several news outlets picked up advertisements of the contest and ran subsequent stories on the contest winners and their health messages. At various stages of the study, the research team also hosted share-back meetings with the YYA advisory board to discuss contest outcomes and actively involve YYA advisors in media coverage, community presentations, and future plans for public display of the YYA-generated art. As a final stage in the dissemination efforts, the research team partnered with Young Arts Arizona Ltd. to display oversized prints of the contest submissions, which will rotate in public galleries across the state, including a location in the legislative hallway of the Arizona State Capitol. 

### 2.5. Evaluation of YYA-Crowdsourced Health Messaging via Contestant Submissions and Community Vote 

To evaluate the acceptability of the crowdsourcing contest and impact of YYA-crowdsourced messages, data metrics were collected on advisors, content submitted to the contest, and community voters. For the purposes of this paper, we will focus our analysis on the number of contest submissions, demographics of contestants and voters, thematic content of submissions, and how that content was reflected upon by community voters. 

An analysis of contestant and voter demographic and behavior change data was conducted in STATA v14, and a thematic analysis of contest submissions and voter reflections on submissions was analyzed in Atlas.Ti version 8.2. As a first step in assessing the utility of materials received and content meaning, the research team gathered submissions into 4 different stylistic types of submission (public service announcement, toolkit items, visual art, and written word). In a subsequent round of open-coding, the research team thematically analyzed submissions by searching for patterns in the messages conveyed within each submission and its accompanying narrative explanation [39]. Similarly, open- and in vivo coding were used to identify emerging themes on the open-ended question posed to voters on the motivations for selecting their vote. 

## 3. Results

### 3.1. Demographics

Table 1 provides demographic data for contestants and community voters. In total, 36 YYAs initiated a contest submission and 23 were completed by the close of the contest deadline. Among completed submissions, roughly half of the contestants (52%) were between 14 and 18 years old and the other half fell between 19 and 24 years of age. An overwhelming majority (78%) of contestants identified as female. The racial-ethnic composition of contestants was as follows: 57% White, 17% Black or African American (AA), 13% Hispanic or Latinx, 9% Asian or Pacific Islander (A/PI), and 4% American Indian or Alaska Native (AI/AN). When we compared these demographics with the racial-ethnic composition of the state, it appears we had a greater share of Black and Asian/PI YYAs in our sample and fewer Hispanic or Latinx YYAs; all other groups were on par with the state averages. Although most contestants who reported on vaccination status reported receiving at least 1 dose of the COVID-19 vaccine, 30% indicated they were either not sure or probably not going to receive a booster vaccine if it was recommended for their age-group.

Turning to demographic data on community voters, 253 individuals visited the voting link and 223 completed a vote. Among the completed votes, 22% of voters were 14–18 years of age; 15% were 19–30 years old; 19% were 31–40 years old; and the remaining 44% were above 40 years of age. Similar to the contestant data, voters were predominantly female (65%). The racial-ethnic composition of voters was as follows: 53% White, 21% Asian or Pacific Islander, 11% Hispanic or Latinx, 5% American Indian or Alaska Native, and 2% Black or African American. These data suggest our community voting sample had a larger share of individuals who identify as Asian/PI and a smaller share who identify as Black/AA or Hispanic/Latinx than represented in state proportions. Different from the YYA contestant data, a larger share of community voters who reported vaccination status indicated they definitely planned to receive a booster vaccine (67%) if it were recommended for their age group. 

### 3.2. Content of YYA-Crowdsourced Messaging

**Figure 1:**
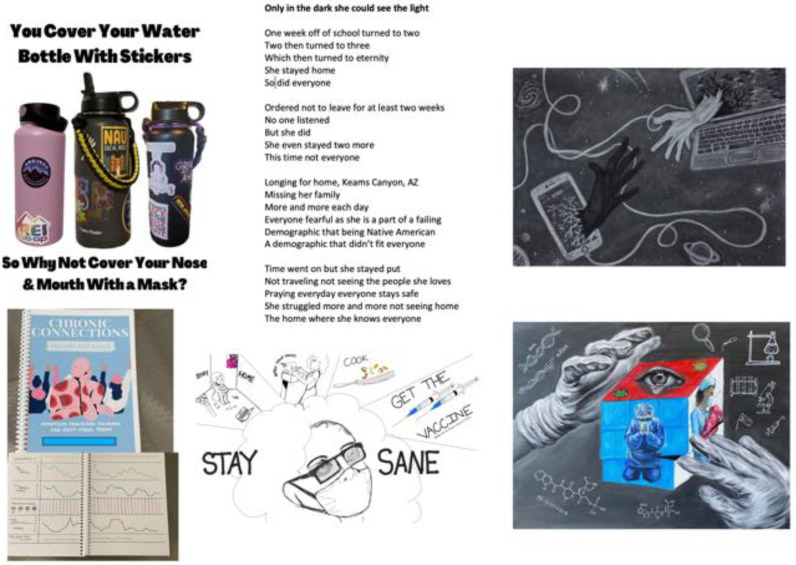
Six examples of YYA-crowdsourced contest submissions. Clockwise: (1) PSA entry about masking; (2) written word entry about the effects of social distancing mitigation on Native American tribal lands; (3) visual art ‘Hold My Hand Through Zoom’; (4) visual art entry to thank health care workers; (5) visual art entry ‘Stay Sane’ highlighting the daily struggles for teens; (6) toolkit entry—a symptom tracker journal for youth with long COVID.

Contest submissions were grouped into categories, intrinsically derived from entry style: public service announcement (6), toolkit items (6), visual arts (7), and written word/poetry (4). The public service announcement category refers to submissions in the form of an infographic poster or short tagline that follow a traditional layout to raise public awareness on health issues. In contrast, the visual arts category includes creative art, primarily visual in nature. Toolkit items are resources generated for use in instruction or managing care, and written word is the art form of poetry and storytelling (see Figure 1 for examples of contest submissions). 

Looking across the stylistic categories, 3 thematic areas dominated the messaging frame of contest submissions: Let us do *our* part to bring this to an end; acknowledge disruptions to daily life and mental wellbeing; and give attention to long COVID in YYAs. Using exemplar quotes and images from contest submissions, hereafter we will provide evidence to illustrate each of these priority health messages created by YYAs for YYAs and their community members.

### 3.3. Theme 1. Let Us Do Our Part to Bring This to an End 

Contest recruitment materials invited YYAs to enter the contest by creating a message to promote vaccination, mask wearing, or raise awareness about another important way the pandemic has affected youth. Unsurprisingly, this prompt resulted in a majority of content submissions having some form of mitigation strategy emphasized in its content. A visual depiction, tagline, or narrative was present on masking in 61% (14) of submissions, whereas the same sort of depictions on vaccination were present in 48% (11) of submissions, and only 2% of submissions took the approach of recommending the full array of mitigation strategies (e.g., social distancing, hand washing). 

When asked to describe their entry, numerous contestants explicitly mentioned their peers as the intended audience. For example, a 14-year-old contestant wrote:

“My motivation was to put out a message about wearing a mask correctly. A lot of people at my school have not been wearing theirs correctly and then the next we they were sick.”

Although a large share of messages urging mitigation efforts were concentrated in the PSA and toolkit style of entry, masking visuals were also prominently featured in visual art and even the focus of a written word entry:

“In my submission is a poem encouraging everyone to wear a mask. I used a long analogy of being a superhero to convey that message, as I feel that when we are kids, we all aspire to special in our own way, and that usually takes the form of wanting to be a superhero. I believe while not everyone can get vaccinated or chose not to, we can all wear a mask to protect the people around us and our loved ones.”

Narrative text describing a contestant’s entry also revealed another important finding on underlying motivations and views on one’s own role in the pandemic response. YYAs rarely centered themselves in mitigation messaging or gave directives towards others, and instead framed messaging in the collective ‘we,’ especially for the purposes of bringing the pandemic to a close. As one 20-year-old contestant wrote: “It is important for my peers to understand that this pandemic is very real. If we don’t do these few simple things, it will never end”. In another example (artwork is featured in Figure 1), a contestant wrote about her visual art, entitled ‘Hold My Hand Through Zoom’:

“[It] explores our need to connect with others despite the dangers we have faced having those connections. We have found ways to adapt and change and fight against the difficulties COVID has given us. By working together, we can save lives and stop the spread and find a new way to connect and understand one another. This is why I want to share my work, so I can do my part.”

In both the ‘Be the Hero’ and ‘Hold My Hand Through Zoom’ examples, we see an emphasis on ‘working together’ and ‘protecting people around us’. YYAs formed messages affirming social responsibility for one another, a practice that aligns with a generation purported to be more socially conscious. 

### 3.4. Theme 2. Disruptions to Daily Life and Mental Health

Disruptions to daily life and the impact on the mental health and wellbeing of YYAs were just as prominently featured in YYA-led messaging as educational messages on COVID-19 mitigation. Nearly half of all submissions included a visual depiction, tagline, or narrative referencing fear/anxiety, stress, or sadness associated with disruptions to daily life. Two prime examples are featured in Figure 1. First is visual art featuring a bulging-eyed young adult surrounded by the words “stay sane” and visuals of multitasking mitigation strategies with staying home and performing household tasks. This contestant wrote in their narrative that their motivation for this piece was to “show my story of what’s happening in my life right now.” Second is an excerpt from a Native American teen who utilized her talents to describe the pride she takes in keeping her community safe by observing lockdowns, but also the toll taken in not being able to visit her tribal lands and be in communion with family and friends. She wrote:

“Until the end which is not near

She will feel deprived of the presence of those she loves

With weeks growing longer and continuing forever

She will stay home so maybe one day she can go home

Go home to be with everyone.”

Related to disruptions to one’s daily routine were losses of time and erasure of key adolescent milestones. For example, one contestant’s poetry submission was titled, ‘The Clock Will Never Stop for You’ and featured this powerful prose “I am not as I began. And every day I sit and think of every second hand. That ticked away the time I had. That ticked away my youth”. We see similar expressions in an excerpt from a 17-year-old’s written word submission ‘If You Only Knew’, which said:

“Conversations weren’t the same anymore.

Every day felt the same.

Wake up, eat, classes, eat, classes, eat, sleep, repeat.

It repeated again and again for weeks upon weeks

Every week was just another broken record.

Happiness started to dwindle away.”

### 3.5. Theme 3. Supports for YYAs Experiencing Long COVID

Although less prominent, a final theme surfacing in contestant submissions was a need for support services to youth who have long COVID, a post-viral condition in which symptoms persist long after the initial infection seems to have cleared. For example, after experiencing long COVID herself, one contestant submitted a symptom-tracking journal she developed to aid other teens who also were afflicted with COVID-19. In her own words:

“In the tracker, I included a space for teens to include their fatigue, a symptom of their choice, their mood, and their goals for the month. At the end of the month, they’ll be able to visualize their symptom progression”.

This example showcases the do-it-yourself spirit embodied by YYAs to utilize their own experiences and talents to contribute to pandemic response efforts, especially in a time period where the focal point remained on emerging support services tailored to adult needs. Through her efforts, the contestant described her own revelations on this contribution: “As my journals made their way across the world, I began to uncover the impact that the symbiosis of writing and science could have on the community as a whole”.

### 3.6. Community Voter Reflections after Viewing YYA-Led Crowdsourced Messaging

During the first dissemination stage of youth-led crowdsourced messages (i.e., the community vote), voters were asked to reflect on whether viewing the submission category influenced their own behavioral intentions on COVID-19 prevention. Example questions included, “Did viewing this submission category influence you to rethink a COVID-19 myth you once believed in” or to “keep wearing a mask indoors?” 

Table 2 summarizes the proportion of respondents who answered in the affirmative to each of the 7 questions. Two notable findings are highlighted here: the lowest frequencies (ranging from 34% to 53%) occurred on the question stem about rethinking a COVID-19 myth you once believed to be true, and the greatest frequencies (76% to 88%) were consistently attached to the question stem on thinking more about how the pandemic has affected youth. All other question stems generated mid-range frequencies, which is evidence suggestive of crowdsourcing approaches’ potential to enact positive behavioral changes among community members. Two possibilities for the lower movement on rethinking a myth are that a majority of submissions never framed their content as addressing myths (and thus respondents perhaps did not perceive submissions as addressing myths), or respondents in this sample were less likely to buy into myths in the first place. The high vaccination rates among community voters would support this second hypothesis. We argue that a majority of voters positively reporting that submissions made them think more about how the pandemic has affected youth bolsters the claim that youth engagement, coupled with a crowdsourcing approach, effectively serve to amplify the voices of YYAs. 

From the open-ended fields on why an individual voted for a particular entry, we found additional evidence to support the claims that a crowdsourcing approach may promote COVID-19 prevention behavioral change and draw attention to YYA experiences and needs. For instance, in reference to the poem on becoming a superhero by masking, one voter wrote: “This speaks so well to the child in all of us. We all remember wanting to be superheroes and now is our chance”.

We also see the power of art in raising awareness on issues important to youth. A teenage voter wrote about the visual of a YYA attempting to ‘stay sane’ while navigating mitigation efforts: “I have personally been experiencing mental illness, so I think it’s important to highlight that it is difficult to stay sane in these difficult times”. Similarly, an adult voter described how another poem about the disruptions of daily life gave her pause: “This entry is so eye opening. We as adults were and continue to be occupied with life and our own experiences through this pandemic, but if we only knew/understood how much youth had to endure during these times”.

In one last exemplary quote, we observe how YYA-led art may also work to evoke empathy and understanding. After viewing the poem by a Native American youth on navigating lockdowns on tribal lands as COVID-19 mitigation, an adult voter who self-identified as White wrote: “I relate to the isolation I felt not being able to see a lot of my family for a long time. It also makes me think about the many ways in which Native tribes were affected by this pandemic”.

## 4. Discussion 

The results from this study demonstrate the opportunities that YYA engagement and crowdsourcing approaches present to investigating the perceptions, needs, and preferred messaging styles of YYAs while also amplifying YYA-voiced solutions during the COVID-19 pandemic response. We found significant interest from YYAs in serving as partners in planning and executing COVID-19 research (84 YYA advisor applications), as well as in trying their own hand at developing health messages aimed at COVID-19 mitigation (23 completed submissions to a brief, time-limited contest). Throughout the course of this research, we were amazed at the level of commitment and leadership displayed by YYA advisors and contestants in promoting the safety of their communities. One YYA advisor described seeing the hardships of her community as her motivation to lead this project: “My family and community were hit hard by COVID-19 this past year. We have lost so many because my people don’t have access to a lot of information concerning COVID-19”. By integrating YPAR and crowdsourcing principles of power-sharing into this research, we are disrupting standard beliefs and practices about who has expertise to create knowledge and who is responsible for public service to others. On an individual level, gains may be seen in YYA self-esteem, critical thinking skills, and perceived control, which are all very relevant to mental health and wellbeing [22,30]. On a societal level, this approach is aligned with promoting inclusivity and ecological approaches to problem-solving. 

One noted limitation of this study, however, was the unevenness in gender across both contestants and community voters. Gender differences have been well documented in perceived risk to COVID-19, such that males perceive less of a risk of contracting COVID-19 and less concern about health consequences compared to females [40,41,42]. In part, we hypothesize that gender differences in perceived risk may also factor into gendered differences regarding participation in COVID-19 research and collective action. Additionally, while racial and ethnic diversity in representation was present among youth advisors and crowdsourcing contestants, greater representation of Hispanic/Latinx and Black/African American voters was not fully achieved among community voters. An additional investigation is warranted to understand the barriers and facilitators to engaging broader diversity in the dissemination of community votes. 

During the first two years of the COVID-19 pandemic, community engagement strategies to mitigate and prevent the spread of COVID-19 inadvertently excluded YYA voices and experiences, and thus excluded a substantial part of the population facing pandemic hardships (e.g., school closures, social isolation, decreased mental health). Our findings from the “It’s Our Turn” crowdsourcing contest suggest that YPAR principles and crowdsourcing contests are a viable way to recover some of that YYA engagement in times of crisis. In our study, crowdsourcing rapidly identified 3 priority COVID-related messages top-of-mind for YYAs in Arizona: (1.) collective action is a necessary, and preferred way to put an end to this pandemic; (2.) disruptions to daily routines are significantly affecting YYAs’ mental health, and (3.) long COVID-19 is also affecting youth and requires youth-specific support services. In addition to gaining insight into YYA perceptions and behaviors on COVID-19 mitigation efforts, the crowdsourcing approach rapidly sourced creative message content from YYAs themselves. As such, not only did the content differ, but also the styling and imagery, to be more reflective of their age bracket. Moreover, a thematic analysis of community voter data showed that when youth-sourced messaging was positioned in front of community members, there was clear evidence that messages were relatable, effective at raising awareness on YYA pandemic experiences and lingering needs, and held utility in their potential to influence behavioral intent among community members with regard to COVID-19 prevention.

By adopting power-sharing principles in this action-oriented research, we believe the resultant process and products better reflect the priorities of youth and young adults: a perspective that had not been prioritized early-on in the pandemic response efforts. As contestant submissions circulate in public galleries across the state in the coming year, our hope is that the YYA-led messages continue to permeate the minds and hearts of all who view their works, including adults in positions of authority who hold substantial influence over the social, economic, and political contexts that continue to shape the lives and lingering issues affecting youth and young adults.

## 5. Conclusions

In short, crowdsourcing is uniquely positioned to rapidly and effectively partner with communities to identify priority topics and develop meaningful strategies to problem-solve. It is important to continue engaging youth and young adults to identify issues important to them, such as pandemic-related trauma or long COVID-19. Indeed, mental health support and access for YYA have yet to be systematically addressed, in part, due to a gap in knowledge of the lingering mental health issues among youth and diverse communities.

## Figures and Tables

**Table 1:** Demographics of Crowdsourcing Contestants and Community Voters.

	Contestants	Voters
	n = 23	n = 223
Age (years)		
14–15	13%	4%
16–18	39%	18%
19–24	48%	8%
25–30		7%
31–40		19%
41–56		27%
57–75		14%
76+		2%
Missing		<1%
Gender		
Male	17%	27%
Female	78%	65%
Non-binary, genderqueer, or gender fluid	4%	3%
Missing		5%
Race/Ethnicity		
White	57%	53%
Black	17%	2%
Hispanic or Latino	13%	11%
American Indian/Alaskan Native	4%	5%
Asian/Pacific Islander	9%	21%
Other		<1%
Missing		7%
Vaccinated against COVID-19 (n=110)	87%	97.27%
Planning to get COVID-19 booster if recommended for age-group? (n = 110)		
Definitely will		67%
Probably will	70%	15%
Not sure yet	17%	16%
Probably not	13%	
Definitely not		3%

**Table 2:** Behavioral intentions of voters after viewing each contest submission category.

	PSA	Toolkit	Visual	Visual	Written Word
Viewing This Submission Category Influenced You to:			(Ages 14–15)	(Ages 16+)	
Voters (n)	172	161	153	153	121
Rethink a COVID-19 myth you once believed in?	34%	47%	46%	47%	53%
Keep wearing a mask indoors?	72%	66%	73%	70%	75%
Return to social distancing?	72%	59%	63%	65%	69%
Get yourself vaccinated?	54%	65%	59%	59%	64%
Get your child(ren) vaccinated?	62%	57%	55%	64%	68%
Talk with friends or family about getting vaccinated?	60%	67%	65%	71%	76%
Think more about how the pandemic has affected youth?	80%	76%	80%	80%	88%

## Data Availability

Not applicable.
